# Identification of crucial genes related to heart failure based on GEO database

**DOI:** 10.1186/s12872-023-03400-x

**Published:** 2023-07-28

**Authors:** Yongliang Chen, Jing Xue, Xiaoli Yan, Da-guang Fang, Fangliang Li, Xuefei Tian, Peng Yan, Zengbin Feng

**Affiliations:** 1 Department of Cardiac Surgery, Affiliated Hospital of Chengde Medical University, 36 Nanyingzi Street, Chengde, Hebei 067000 China; 2 Experimental Center of Morphology, College of Basic Medicine, Chengde Medical University, Chengde, Hebei China

**Keywords:** Heart failure, Diagnosis, Support Vector Machine, Random Forest, Artificial neural network

## Abstract

**Background:**

The molecular biological mechanisms underlying heart failure (HF) remain poorly understood. Therefore, it is imperative to use innovative approaches, such as high-throughput sequencing and artificial intelligence, to investigate the pathogenesis, diagnosis, and potential treatment of HF.

**Methods:**

**First**, we initially screened Two data sets (GSE3586 and GSE5406) from the GEO database containing HF and control samples from the GEO database to establish the Train group, and selected another dataset (GSE57345) to construct the Test group for verification. Next, we identified the genes with significantly different expression levels in patients with or without HF and performed functional and pathway enrichment analyses. HF-specific genes were identified, and an artificial neural network was constructed by Random Forest. The ROC curve was used to evaluate the accuracy and reliability of the constructed model in the Train and Test groups. Finally, immune cell infiltration was analyzed to determine the role of the inflammatory response and the immunological microenvironment in the pathogenesis of HF.

**Results:**

In the Train group, 153 significant differentially expressed genes (DEGs) associated with HF were found to be abnormal, including 81 down-regulated genes and 72 up-regulated genes. GO and KEGG enrichment analyses revealed that the down-regulated genes were primarily enriched in organic anion transport, neutrophil activation, and the PI3K-Akt signaling pathway. The upregulated genes were mainly enriched in neutrophil activation and the calcium signaling. DEGs were identified using Random Forest, and finally, 16 HF-specific genes were obtained. In the ROC validation and evaluation, the area under the curve (AUC) of the Train and Test groups were 0.996 and 0.863, respectively.

**Conclusions:**

Our research revealed the potential functions and pathways implicated in the progression of HF, and designed an RNA diagnostic model for HF tissues using machine learning and artificial neural networks. Sensitivity, specificity, and stability were confirmed by ROC curves in the two different cohorts.

**Supplementary Information:**

The online version contains supplementary material available at 10.1186/s12872-023-03400-x.

## Introduction

Heart failure (HF) is caused by several factors, including systolic and/or diastolic dysfunction, cardiac pumping dysfunction, and end-stage of cardiovascular diseases, which seriously threaten human health [1]. In recent years, with advancements in social medicine and population aging, the incidence of cardiac dysfunction in patients with various cardiovascular diseases has gradually increased, and the incidence of HF has continued to rise, posing a serious threat to public health [2]. According to epidemiological statistics, the global prevalence of HF is 1–2%, with over 10% of affected individuals above the age of 70 years [3]. Between 2013 and 2016, an estimated 6.2 million American adults over the age of 20 years suffered from HF [4]. According to the latest survey report, the life-long risk of HF increases after 45 years of age and varies from 20 to 45%, depending on race and ethnicity [5]. Therefore, the prevention and treatment of HF has become the highest priority for medical professionals.

Drug treatment is the primary treatment modality for patients with HF. A new era of neuroendocrine inhibitor treatment for HF began in 1987, when CONSENSUS successfully confirmed that treatment with angiotensin-converting enzyme inhibitors reduced the mortality rate in patients with HF by 27% [6]. Subsequently, other potent drugs, such as angiotensin receptor neprilysin inhibitor (ARNI) [7] and sodium-glucose cotransporter 2 inhibitors (SGLT2i) [8, 9], have developed to reduce mortality in patients with HF. With advances in clinical management, targeted therapies are being implemented in the cardiovascular field, and are expected to represent a significant breakthrough in the treatment of patients with HF [10]. Various factors can contribute to myocardial injury and subsequent aseptic inflammation of the myocardium [11]. If a pathogenic infection is present, it leads to inflammatory damage to the myocardium [12], activates inflammatory factors, and finally results in myocardial fibrosis [13]. Inflammatory reactions are involved in both the onset and progression of HF. Most researchers believe that hemodynamic disorders, tissue injury and gastrointestinal mucosal ischemia in patients with HF can directly or indirectly activate the immune system in vivo, increasing circulating inflammatory cytokine levels. These cytokines activate the target cells by interacting with specific receptors on the cell membrane, thereby triggering a systemic inflammatory response [14, 15]. Therefore, finding an effective method of diagnostic and therapeutic strategy, elucidating the molecular biological mechanisms of pathogenesis, and inhibiting damage caused by inflammatory reactions, are crucial for inhibiting and delaying the progression of HF.

Biomarkers are biological molecules found primarily in the blood, other bodily fluids, or tissues, and are usually composed of DNA, RNA, microRNA, epigenetic modification, or antibodies. They possess hypersensitivity, specificity, and positive diagnostic value for diseases [16, 17]. HF is a complex pathophysiological process, involving multiple factors [18], however, it can be predicted using a single gene [19]. Bioinformatics is a high-throughput technique that can be used to screen multiple databases to identify potential pathological biomarkers for various diseases [20]. In recent years, the development and application of DNA microarrays and next-generation sequencing technologies, have enabled simultaneous analysis of thousands of genes in different disease samples. Therefore, the use of biomarkers for diagnosis, prognosis, and personalized medical services has increased. Numerous studies have been conducted on biomarkers for the diagnosis of HF. B-type natriuretic peptide (BNP) was one of the earliest biomarkers used to diagnose acute HF [21]. The plasma concentration, stability, and diagnostic value of Nt-proANP and Nt-proBNP are higher in patients with chronic HF [22, 23]. Studies have shown that the combined determination of adiponectin and NT-proBNP is more accurate than that of NT-proBNP alone [24]. Other diagnostic biomarkers, such as miR-302b-3p [25], Soluble ST2 [26], and Gal-3 [27], have recently been identified.

Since the beginning of the 21st century, artificial intelligence (AI) has progressively permeated all aspects of our existence, particularly in the medical field [28]. With continued exploration of the potential of artificial intelligence, AI-based clinical research will result in a paradigm shift in medical practice, thus significantly improving the survival rate of many diseases including cancer [29]. Currently, the diagnosis of HF is primarily based on the clinical signs and symptoms of patients, with echocardiography and chest radiography serving as the most common auxiliary tests. However, these examinations are not accurate during the intermediate and late phases of the disease and lack clinical specificity and sensitivity. Therefore, exploring a reliable diagnostic approach to reduce mortality and improve the prognosis of patients with HF is critical. In this study, we first identified the characteristic abnormal genes associated with HF using machine learning, and then constructed and validated a prediction model using an artificial neural network.

## Methods

### Data acquisition

We accessed the available datasets from the GEO website (https://www.ncbi.nlm.nih.gov/geo/) to construct HF and NFD (non-failure donor) cohorts. The Train group included 16 cases without HF, 86 cases with dilated cardiomyopathy (CMP), and 108 cases with ischemic heart disease from GSE5406 dataset [30], as well as 13 cases of dilated cardiomyopathy and 15 cases of non-HF GSE3586 dataset [31]. To verify the reliability and stability of the artificial neural network model more thoroughly, we included 96 cases with ischemic heart disease, 84 cases with dilated cardiomyopathy (CMP), and 139 cases without HF in the Test group from the GSE57345 dataset [32].

### Analysis of differential gene expressions and functional enrichment in HF

The R package “limma” was used to identify differentially expressed genes (DEGs) in HF, and “heatmap” was used to plot heat and volcano maps. Genes with |logFC| > 0.6 (|fold change| ≥ 1.5) and false discovery rate (FDR) < 0.05 were considered statically significant. Subsequently, we performed GO and KEGG enrichment analysis [33] using R packages “org.Hs.eg.db”(3.14.0), “clusterProfiler”(4.0), “ggplot2”(3.3.5) and “enrichment plot”. For further biological insights into the DEGs, we conducted bioinformatics analysis of Reactome, WikipathwayCancer and Metascape analysis using WebGestalt 2019 website (http://www.webgestalt.org/) and Metascape (version 3.5; http://metascape.org/), while protein-protein interaction modes were obtained from the String website (version 11.5; https://cn.string-db.org/).

### Identification of disease-specific genes and construction of an artificial neural network

We then conducted a random-forest analysis using the “randomForest” package (version 4.6) and filtered the DEGs to identify the nodes with the lowest cross-validation errors. The parameter settings were seed = 123,456 and ntree = 500. Homologous genes acquired using the above two approaches were identified as HF-specific genes. Disease signature genes were visualized using the “limma” and “pheatmap” packages (version 3.5.3), and the samples were clustered according to their expression. To eliminate batch effects between cohorts, we scored the DEGs based on their expression relative to the median value: upregulated genes were assigned a score of 1 for values, greater than the median value, otherwise, they were scored 0. When this gene was down-regulated, the score followed the opposite pattern. We constructed an artificial neural network to diagnose HF using gene scores. The neural network consisted of three layers, an input, a hidden, and an output layer. The R package used in this step was “NeuralNetTools”, and the seed was set to 12,345,678.

### Evaluation of the artificial neural network model

The same approach was used to test and validate the gene cohort, and to evaluate the diagnostic accuracy of the HF model. To evaluate the efficiency of the artificial neural network model, we plotted ROC curves for the two cohorts using the “pROC” package (1.15.3). In the ROC curve, the horizontal scale denoted the false positive rate, representing “1-Specificity”, and the vertical scale denotes the true positive rate, representing “Sensitivity”. The area under the curve (AUC) represented the accuracy of the model, which was our primary focus.

### The immunological milieu of HF

The CIBERSORT algorithm (https://cibersort.stanford.edu/runcibersort.php) for immune cell infiltration was used to quantify 22 immune cells, and the results were filtered using a p-value < 0.05. The analysis was performed using the R packages “e1071”, “preprocessCore” and “CIBERSORT.R”. Based on these results, we calculated the correlation between immunocytes. The “corrplot” package (version 0.92) visually displayed immune cell contents and predicted their correlation. Finally, we measured the distribution of immunocytes, which differed between cases with or without HF.

## Results

### Identification of DEGs and functional enrichment analysis

**Fig. 1:**
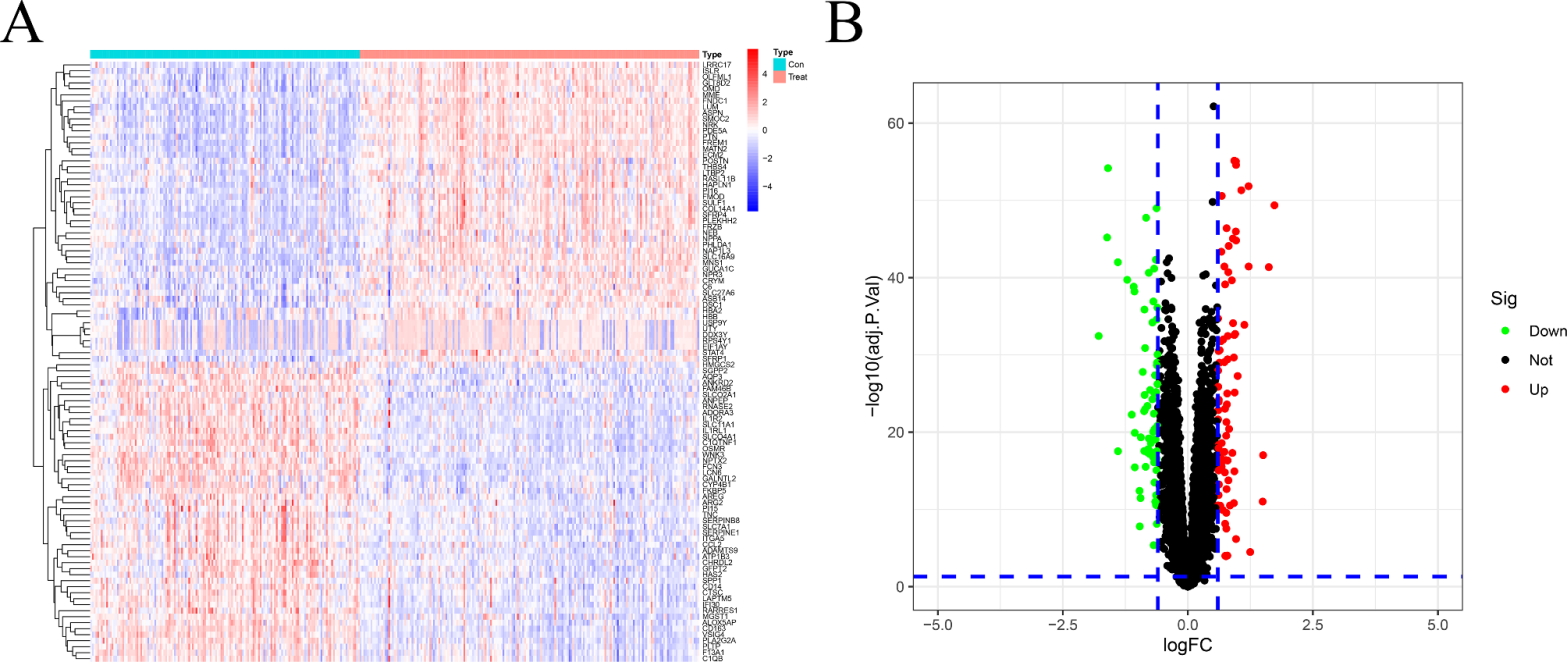
Genome-wide identification of differentially expressed genes of HF. (**A**) The heatmap of DEGs in Train group. (**B**) The volcano plots of DEGs.

**Fig. 2:**
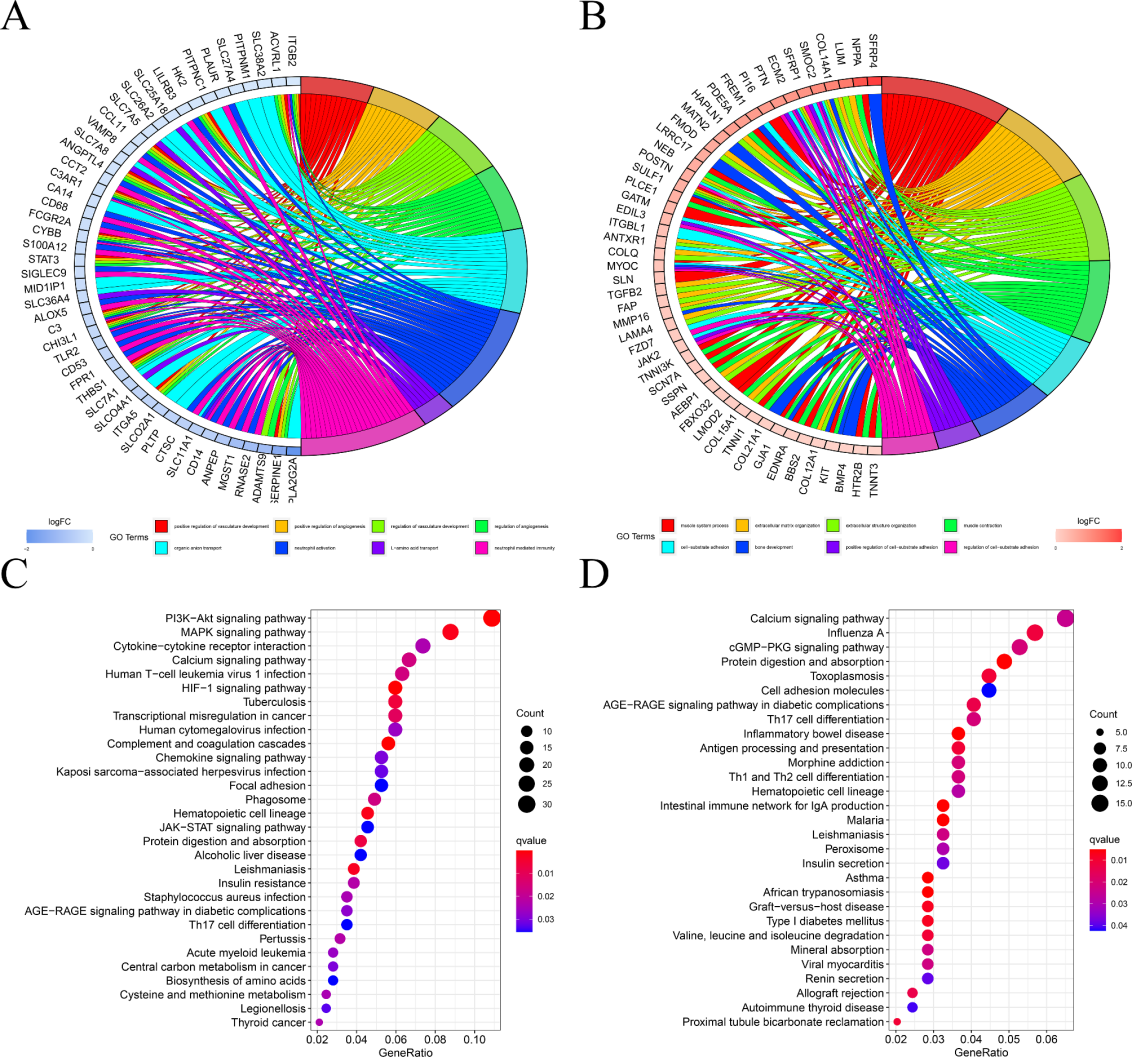
Functional enrichment analyses of DEGs for HF. (**A**) Chord diagrams of GO terms belonging to the top 81 down-regulated genes of HF. (**B**) Chord diagrams of GO terms belonging to the top 72 up-regulated genes of HF. (**C**) Chord diagram of KEGG enrichment pathway of the first 81 down-regulated genes in HF. (**D**) Chord diagram of KEGG enrichment pathway of the first 72 down-regulated genes in HF.

After setting the parameters |logFC| > 0.6 and FDR < 0.05, differential expression analysis of the GEO dataset revealed 153 differentially expressed genes, of which 81 were down-regulated and 72 were up-regulated (Fig. 1A and B, **Additional File Table 1**). For these DEGs, GO enrichment analysis showed that the 81 down-regulated genes were primarily associated with the positive regulation of vascular development, angiogenesis, neutrophil activation, L-amino acid transport, and neutrophil-mediated immunity (Fig. 2A). The up-regulated 72 genes were primarily involved in muscle system processes, extracellular matrix organization, extracellular structure organization, muscle contraction, and cell-substrate adhesion (Fig. 2B). KEGG enrichment analysis indicated that the down-regulated 81 genes were mainly associated with the PI3K-AKT signaling pathway, MAPK signaling pathway, Cytokine-cytokine receptor interaction, Calcium signaling pathway, HIF-1signaling pathway, Chemokine signaling pathway, Focal adhesion, JAK-STAT signaling pathway, AGE-RAGE signaling pathway in diabetic complications, Th17 cell differentiation, and amino acids biosynthesis (Fig. 2C). The up-regulated 72 genes were mainly involved in the Calcium signaling pathway, cGMP-PKG signaling pathway, AGE-RAGE signaling pathway in diabetic complications, Th17 cell differentiation, Th1, and Th2 cell differentiation, Peroxisome, Valine, leucine, isoleucine degradation, and renin secretion pathways (Fig. 2D).

### Prediction of the function and disease spectrum of HF-related factors

**Fig. 3:**
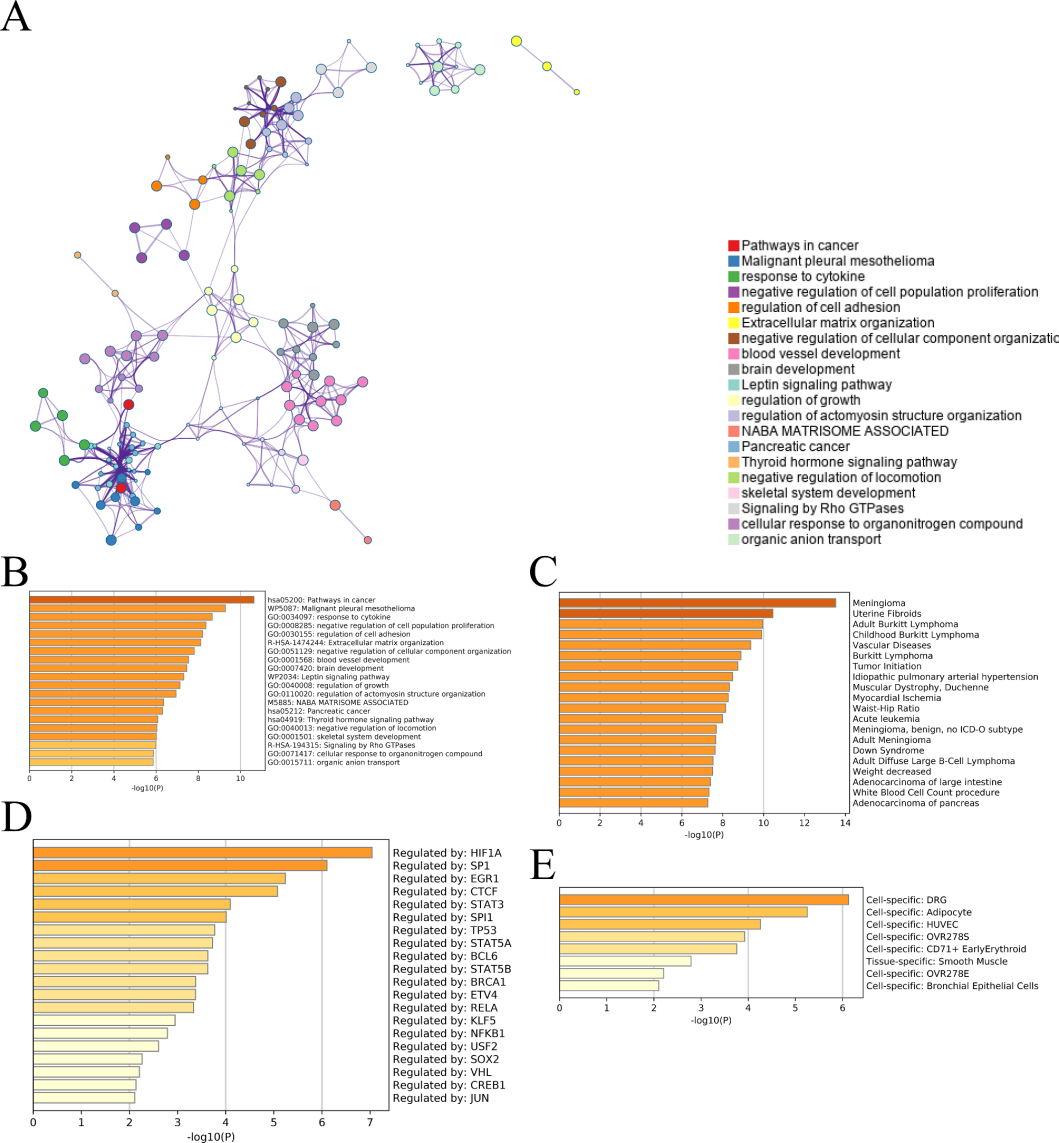
Prediction of function and disease spectrum of factors related to HF. (**A**) Metascenario analysis provides an overview of network diagrams. (**B**) List factors associated with HF. (**C**) The HF-related diseases through disease prevalence were predicted. (**D**) HF-related transcription factors from the perspective of epigenetics were screened. (**E**) The genes in cells were listed

The meta-scene analysis provided an overview of the network diagram (Fig. 3A). The nodes in this network represented the functions or pathways. The higher the similarity between the two nodes, the more genes were shared between the two functions or pathways. Figure 3B lists the HF-related factors, such as regulation of cell adhesion and vascular development, while Fig. 3C, HF-related diseases are predicted based on disease prevalence, such as idiopathic pulmonary arterial hypertension and myocardial ischemia. Additionally, Fig. 3D screens HF-related transcription factors, including HIF1A, SP1, EGR1, and CTCF from an epigenetic perspective. Figure 3E depicts the function of these genes within specific cells.

### Network analysis of protein-protein interactions

**Fig. 4:**
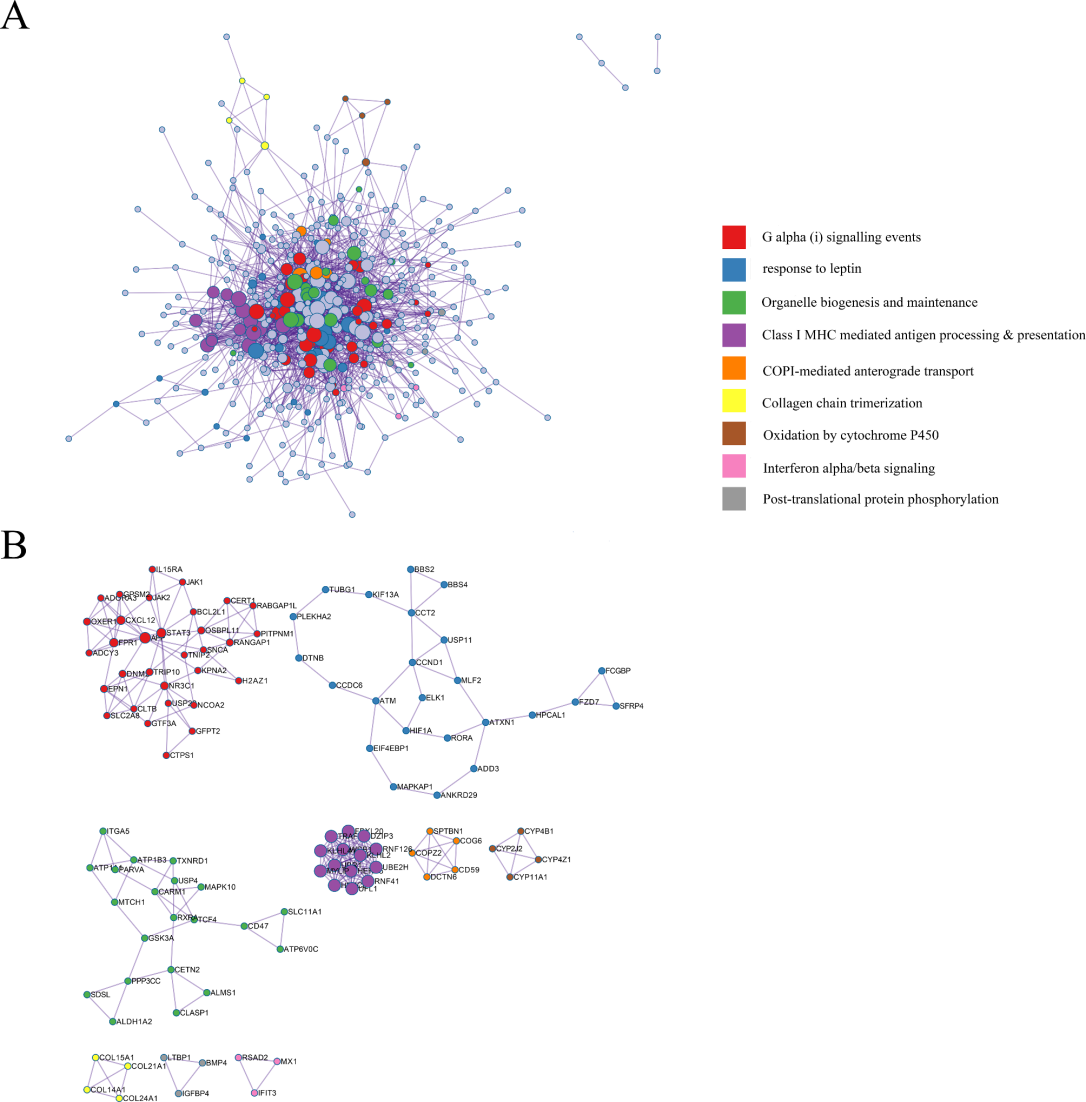
The Metascape analyses the protein-protein interaction. (**A**) Pathway and process enrichment analysis of HF. (**B**) The sub-module analysis of protein-protein Interaction

Protein-protein interaction (PPI) enrichment of the HF-related genes was analyzed using Metascape algorithm. In the network diagram, nodes represented genes or proteins, and nodes with the same color represent genes or proteins with related functions. The connection between the two nodes indicated a protein-protein interaction between the two genes (Fig. 4A), and Fig. 4B shows the correlation between functionally different genes or proteins.

### Selection of disease-specific genes and prediction model for the HF

**Fig. 5:**
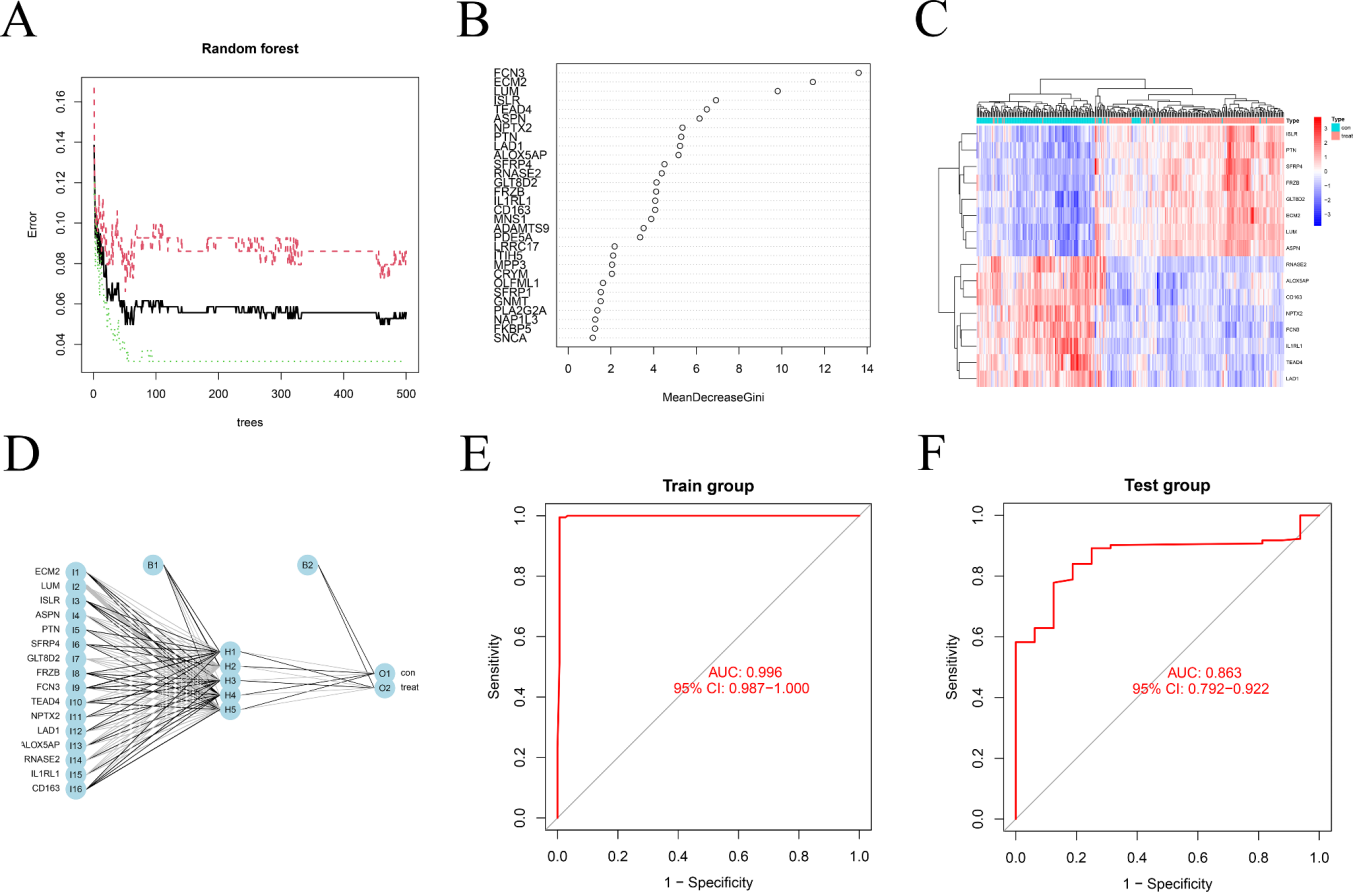
Identification of characteristic genes of HF by machine learning and the construction of diagnosis signature by an artificial neural network. (**A**) The construction of RandomForest. (**B**) Identification of HF signature genes based on significance scores. (**C**) The heatmap of CRC characteristic genes. (**D**) Schematic view of the artificial neural network. (**E**) The ROC curves demonstrate the diagnostic performance of the artificial neural networks for HF in Train Group (GEO). (**F**) Test Group (HF of TCGA)

Figure 5 A illustrates the random forest algorithm, with the X- and y-axes representing the number of trees and cross-validation error, respectively. The black lines indicate the error values for all samples. During cross-validation, we identified the point with the minimum error. After locating this point, the number of trees corresponding to this point, which was the lowest point on the black line, was determined. Then Fig. 5B was created, the Y-axis represented the gene name, and the X-axis represented the importance score of the gene. The gene was considered more important if the score was higher. Genes with scores higher than 4 were selected for subsequent analysis. The heat map (Fig. 5C) showed the aggregation of genes, indicating the pathogenic nature of the genes detected in random forest trees. Figure 5D depicts the construction of a neural network model based on gene scores, where the input layer comprising genes for multiple diseases was linked to the hidden layer displaying disease-related genes according to their obtained scores and weights. We observed that there were five nodes in the hidden layer. Based on these five nodes and their respective weights, we obtained the output layer, which was the attribute of the sample. The accuracy of the model was further evaluated by constructing ROC curves. The accuracy of train group and test group was 0.993 and 0.995, respectively. Figure 5E, F clearly show that the areas under the ROC curves are 0.996 and 0.863, respectively. The AUC values were greater than 0.75, indicating that our diagnostic model was accurate, reliable, and unaffected by alterations in the cohort group. The precision, recall, and F1 score of Train group were 0.957, 0.963, and 0.945, respectively. The precision, recall, and F1 score of Test group were 0.893, 0.826, and 0.842, respectively.

### The immune microenvironment of HF

**Fig. 6:**
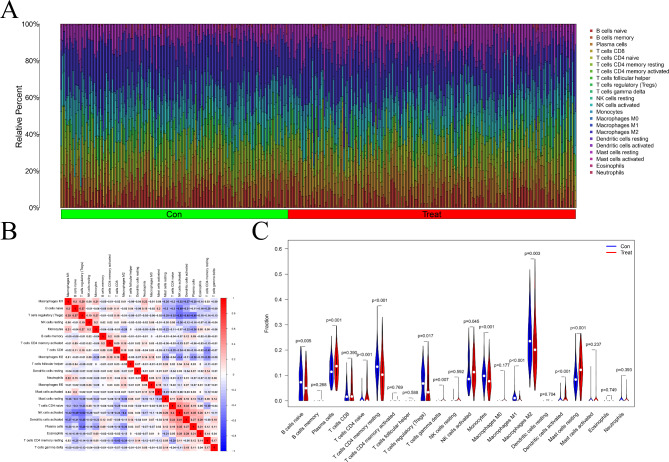
The immune microenvironment of HF. (**A**) Histogram of 22 kinds of immune cells in HF patients and normal controls. (**B**) The correlation between various immune cells of HF patients. (**C**) Violin chart of differences of individual immune cells

The histogram in Fig. 6A displays the presence of 22 distinct immune cell types. We assessed their correlations by determining the infiltration of immune cells. The results are shown in Fig. 6B, with numbers representing the correlation coefficient, red indicating a positive correlation, and blue indicating a negative correlation. The highest positive correlation coefficient between activated dendritic cells activated and NK cells was 0.41, and the highest negative correlation coefficient between activated NK cells and regulatory T cells was − 0.52. Then the immune cell fractions were compared between the groups, and the results revealed significant differences in naïve B cells, plasma cells, CD4 naïve T cells, CD4 memory-activated T cells, regulatory T cells, γδ T cells, activated NK cells, monocytes, M2 macrophages, activated DC, and resting mast cells (*P* < 0.05, Fig. 6C).

## Discussion

The mechanism of HF is complex and has not yet been fully elucidated at present. Research suggests that abnormal vascular microcirculatory metabolism [34], aberrant expression of multiple inflammatory markers [35, 36], immune responses [37], and abnormal expression of metabolic proteins are closely associated with heart failure [38]. HF is the leading cause of death from cardiovascular disease. Various RNAs and genes are involved in regulating cellular activities of vascular smooth muscle cells then affecting cardiovascular disease [39, 40]. For instance, inflammatory cytokines IL-6 and TNF-α could be the main targets of miR-296a, and their expression was abnormal in peripheral blood mononuclear cell of patients with coronary artery disease [40]. Despite significant advances in the treatment of HF in recent years, the 5-year survival rateremains approximately 50% [41], and the prognosis is still poor. Therefore, early diagnosis and treatment of patients are crucial for reducing the incidence and mortality of HF patients. In this study, a HF model consisting of 16 characteristic genes was constructed using machine learning and artificial intelligence based on high-throughput sequencing data from public databases. The model demonstrates high sensitivity and specificity for screening purposes, to prevent HF.

HF transcription factors involved in genome regulation have been proposed as putative epigenetic mechanisms. Zhao and his team workers [42] demonstrated that MIAT silencing reduces the incidence of HF by activating the PI3K/Akt signaling pathway. The present study found that the PI3K-Akt signaling pathway is down-regulated in HF. Consistent with the findings of this study, previous research has demonstrated that the JAK/STAT signaling pathway mediates the inflammatory response, left ventricular remodeling, and myocardial ischemia-reperfusion injury, via the downregulation of genes enriched in the JAK-STAT signaling pathway [43, 44]. Furthermore, the downregulated GO and KEGG genes were enriched in the HIF-1 signaling pathway, Th17 cell differentiation, organic anion transport, neutrophil activation, and other pathways and functions. Studies [45] have revealed that CaMK II oxidative activity is significantly increased in patients with HF, thereby activating the calcium signaling pathway, which is consistent with the results of this study. Moreover, downregulated GO and KEGG genes were enriched in the cGMP-PKG signaling pathway, AGE-RAGE signaling pathway in diabetic complications, muscle system processes, and extracellular matrix organization. Th17 cell differentiation was enriched in both up-regulated and down-regulated genes. Research [46–48] has highlighted that Th17 cells can produce IL-17 and IL-22, which are key effector cytokines. IL-17 is an effective inducer of matrix metalloproteinase-1 (MMP-1) in human cardiac fibroblasts, which may have potential implications in cardiac fibrosis, remodeling, and heart failure through various pathways.

We identified disease-specific genes for HF using a random forest algorithm in machine learning to facilitate the integration of neural network models. The artificial neural network method, which has been extensively applied to cancer diagnosis and treatment models, was used to construct a diagnostic model of HF [49]. The prediction model of rectal cancer-related microsatellite instability (MSI) established by Stanford University [50] successfully predicted MSI by identifying the whole-glass scanning image (WSI) of HE staining. Moreover, the DeepLabV3 + semantic segmentation model exhibits good feature extraction and semantic image segmentation abilities. ResNet50, a classical image-classification model, has been widely used for target classification and other fields [51, 52]. Artificial neural network models have been applied to lung cancer [53] and breast cancer [54]. This is the first time that an artificial neural network approach has been used to develop a heart failure disease model. Our diagnostic signal comprised of 16 genes (ECM2, LUM, ISLR, ASPN, PTN, SFRP4, GLT8D2, FRZB, FCN3, TEAD4, NPTX2, LAD1, ALOX5AP, RNASE2, IL1RL1, CD163). Currently, there are no studies on the direct correlation between the ECM2, GLT8D2, NPTX2, LAD1 genes and HF, however, evidence suggests their potential association with HF, may become potential biomarkers for HF diagnosis in the future [18, 55–57]. For instance, ECM2 was related to immune process and could serve as a target for immunotherapy for glioma [58, 59]. Consistently, the present study observed that HF-related genes, including ECM2 and CD163, were associated with immune cells. CD163, a receptor for tumor necrosis factor-like weak apoptosis-inducing factor (TWEAK), may serve as a novel marker of HF. Studies demonstrated its anti-inflammatory, antioxidant and cardiovascular-protective effects [60–62]. Furthermore, serum TWEAK levels are significantly higher in patients with HF than in healthy individuals [63]. ILIRL1 could be induced by cardiomyocyte stretch, and might reflect inflammation and hemodynamic stress in HF [64]. HIF-α is a key factor mediating the relationship between obesity and HF through affecting fibrosis and inflammation in adipose tissue [65]. This demonstrates the effectiveness of gene screening in this study, and the significance of these genes in the diagnosis, treatment, and prognosis of various diseases.

The pathological basis of HF is ventricular remodeling, specifically myocardial hypertrophy and fibrosis, which results from hemodynamic overload [66]. The JAK/STAT signaling pathway [44], reactive oxygen species (ROS) generation [67], calcium overload [68], Th17 cells, PI3K/AKT signaling pathway [69], and MAPK signaling pathway have all been implicated in the pathophysiology of HF. These findings are consistent with the results of the GO/KEGG enrichment pathway. In addition, cell adhesion molecules are also involved in the process of HF. A previous study noted that focal adhesion kinase-related pathways may be inhibited in metformin-treated vascular smooth muscle cells then retard the progression of vessel stenosis [70]. Heart failure is frequently associated with immune activation and inflammatory responses. As important inflammatory mediators, chemokines, exert chemotactic effects on various target cells, including vascular endothelial cells, which can contribute to the development of HF [71]. Studies have suggested interactions between myocardial cells and the microvascular system. Persistent pathological overload leads to cardiac maladaptation and remodeling, resulting in HF. At the same time, cellular senescence affects the cardiac regeneration and recovery in patients with ischemic heart disease. The study of the differential expression of metabolic proteins in patients with HF can enable a better understanding of the occurrence of HF, particularly the crucial role of angiogenesis factors [38]. Echocardiography is the most commonly used method for diagnosing heart failure. The disadvantage is that patients have organic lesions, and the artificial neural network is primarily calculated based on the scores of various factors, and after which the diagnosis is made. Neural networks have proven to be highly reliable in the diagnosis of HF, which is the first time this approach has been used in this context.

In addition to establishing early diagnostic models, we investigated the immune microenvironment of HF. Studies have shown that the pathological mechanisms underlying heart failure include inflammatory immune responses and inflammatory cell infiltration [37]. This study found a significant increase in dendritic cells (DCs) in cases of heart failure. DCs express MHC II, making them a unique cell group that presents antigens to T cells. DCs also secrete numerous growth factors and cytokines to modulate immune responses and inflammation [72]. Studies on myocardial infarction suggest that the eventual outcome of DCs activity depends on the subset of DCs involved and the type of effector cells that are subsequently recruited. Ideally, the activation of conventional dendritic cells, should increase the activity of tolerant dendritic cells to rapidly reduce inflammation [73]. NK cells are positively correlated with DCs proliferation. Activation of NK cells depends on the balance between activation and inhibitory signals from target cells [73]. In acute myocardial infarction, studies have shown that NK cells promote dendritic cell differentiation by releasing cytokines, thus forming a positive feedback pathway and influencing ventricular remodeling [74]. The primary pathology of HF is ventricular remodeling. Although there is no direct evidence of association between NK cells and HF, it can be confirmed that NK cells are increased in the pathophysiology of HF, which is consistent with the findings of this study. In contrast, investigations into cardiovascular diseases have revealed that the number of regulatory T cells is reduced. Regulatory T cells are a subset of CD4 + T cells with unique immunoregulation abilities that maintain immune homeostasis in the body, primarily through cell contact and the release of inhibitory cytokines (such as IL-10 and TGF-β1) [75]. Consistent with the results of this study, there may be a negative correlation between NK and regulatory T cells. However, this study had some limitations. First, this was a retrospective analysis, using datasets retrieved from the public database. Moreover, we only verified the predictive performance of HF, treatment and prognosis require further investigation. Additionally, experimental and clinical studies are necessary to validate the results of this study and to assess their implications for the treatment and prognosis of HF.

## Conclusions

We developed an accurate HF diagnostic model using machine learning and an artificial neural network. Despite disparities between patient cohorts, this signature is still effective and can be used for personalized disease prediction and precision medicine. In addition, immune regulation plays crucial role in the progression of HF, and our results have potential implications for the use of immunotherapies to treat HF patients in the later stage. Substantive and scientific validation of these findings warrants large-scale prospective clinical trials and experimental studies.

## Electronic supplementary material

Below is the link to the electronic supplementary material.

Additional File Table 1: The list of up-regulated and down-regulated DEGs

## Data Availability

Publicly available datasets were analyzed in this study. This data can be found here: GEO website: https://www.ncbi.nlm.nih.gov/geo/. WebGestalt 2019: http://www.webgestalt.org/. Metascape (version 3.5): http://metascape.org/. R software (version 4.0.4): https://www.R-project.org. String website (version 11.5): https://cn.string-db.org/. CIBERSORT algorithm: https://cibersort.stanford.edu/runcibersort.php.

## References

[1] Cleland JG, Khand A, Clark A (2001). The heart failure epidemic: exactly how big is it?. Eur Heart J.

[2] Tschöpe C, Kherad B, Klein O, Lipp A, Blaschke F, Gutterman D (2019). Cardiac contractility modulation: mechanisms of action in heart failure with reduced ejection fraction and beyond. Eur J Heart Fail.

[3] Kuschyk J, Rudic B, Liebe V, Tülümen E, Borggrefe M, Akin I (2018). [Cardiac contractility modulation for treatment of chronic heart failure]. Herzschrittmachertherapie Elektrophysiologie.

[4] Virani SS, Alonso A, Benjamin EJ, Bittencourt MS, Callaway CW, Carson AP (2020). Heart Disease and Stroke Statistics-2020 update: a Report from the American Heart Association. Circulation.

[5] Tsao CW, Aday AW, Almarzooq ZI, Alonso A, Beaton AZ, Bittencourt MS (2022). Heart Disease and Stroke Statistics-2022 update: a Report from the American Heart Association. Circulation.

[6] Effects of enalapril on (1987). Mortality in severe congestive heart failure. Results of the Cooperative North Scandinavian Enalapril Survival Study (CONSENSUS). N Engl J Med.

[7] McMurray JJ, Packer M, Desai AS, Gong J, Lefkowitz MP, Rizkala AR (2014). Angiotensin-neprilysin inhibition versus enalapril in heart failure. N Engl J Med.

[8] Bhatt DL, Szarek M, Steg PG (2021). Sotagliflozin in patients with diabetes and recent worsening. Heart Fail.

[9] McDonald M, Virani S, Chan M, Ducharme A, Ezekowitz JA, Giannetti N (2021). CCS/CHFS heart failure guidelines update: defining a New Pharmacologic Standard of Care for Heart failure with reduced ejection fraction. Can J Cardiol.

[10] Abdellatif M, Trummer-Herbst V, Koser F, Durand S, Adão R, Vasques-Nóvoa F et al. Nicotinamide for the treatment of heart failure with preserved ejection fraction. Sci Transl Med. 2021;13(580). 10.1126/scitranslmed.abd7064 PMC7611499 33568522

[11] Kong P, Christia P, Frangogiannis NG (2014). The pathogenesis of cardiac fibrosis. Cell Mol Life Sci.

[12] Yajima T, Knowlton KU (2009). Viral myocarditis: from the perspective of the virus. Circulation.

[13] Frangogiannis NG (2008). The immune system and cardiac repair. Pharmacol Res.

[14] Chow SL, Maisel AS, Anand I, Bozkurt B, de Boer RA, Felker GM (2017). Role of biomarkers for the Prevention, Assessment, and management of Heart failure: a Scientific Statement from the American Heart Association. Circulation.

[15] Murphy SP, Kakkar R, McCarthy CP, Januzzi JL (2020). Jr. Inflammation in Heart failure: JACC state-of-the-art review. J Am Coll Cardiol.

[16] Bray F, Jemal A, Grey N, Ferlay J, Forman D (2012). Global cancer transitions according to the Human Development Index (2008–2030): a population-based study. Lancet Oncol.

[17] Cheung HW, Cowley GS, Weir BA, Boehm JS, Rusin S, Scott JA (2011). Systematic investigation of genetic vulnerabilities across cancer cell lines reveals lineage-specific dependencies in ovarian cancer. Proc Natl Acad Sci USA.

[18] Guo Y, Ning B, Zhang Q, Ma J, Zhao L, Lu Q (2022). Identification of hub diagnostic biomarkers and candidate therapeutic drugs in Heart failure. Int J Gen Med.

[19] Zhang K, Qin X, Wen P, Wu Y, Zhuang J (2021). Systematic analysis of molecular mechanisms of heart failure through the pathway and network-based approach. Life Sci.

[20] Lam KK, Thean LF, Cheah PY (2021). Advances in colorectal cancer genomics and transcriptomics drive early detection and prevention. Int J Biochem Cell Biol.

[21] Kevin Rogers R, Stehlik J, Stoddard GJ, Greene T, Collins SP, Peacock WF (2009). Adjusting for clinical covariates improves the ability of B-type natriuretic peptide to distinguish cardiac from non-cardiac dyspnoea: a sub-study of HEARD-IT. Eur J Heart Fail.

[22] Roberts E, Ludman AJ, Dworzynski K, Al-Mohammad A, Cowie MR, McMurray JJ (2015). The diagnostic accuracy of the natriuretic peptides in heart failure: systematic review and diagnostic meta-analysis in the acute care setting. BMJ (Clinical research ed).

[23] Dao Q, Krishnaswamy P, Kazanegra R, Harrison A, Amirnovin R, Lenert L (2001). Utility of B-type natriuretic peptide in the diagnosis of congestive heart failure in an urgent-care setting. J Am Coll Cardiol.

[24] Dai Z, Zhang Y, Ye H, Zhang G, Jin H, Chen Z (2018). Adiponectin is valuable in the diagnosis of acute heart failure with renal insufficiency. Experimental and therapeutic medicine.

[25] Li G, Song Y, Li YD, Jie LJ, Wu WY, Li JZ (2018). Circulating miRNA-302 family members as potential biomarkers for the diagnosis of acute heart failure. Biomark Med.

[26] Huang DH, Sun H, Shi JP (2016). Diagnostic value of Soluble suppression of Tumorigenicity-2 for heart failure. Chin Med J.

[27] Stoica A, Şorodoc V, Lionte C, Jaba IM, Costache I, Anisie E (2019). Acute cardiac dyspnea in the emergency department: diagnostic value of N-terminal prohormone of brain natriuretic peptide and galectin-3. J Int Med Res.

[28] Huang S, Yang J, Fong S, Zhao Q (2020). Artificial intelligence in cancer diagnosis and prognosis: Opportunities and challenges. Cancer Lett.

[29] Zoppo F, Gagno G, Perazza L, Cocciolo A, Mugnai G, Vaccari D (2021). Electroanatomic voltage mapping and characterisation imaging for “right ventricle arrhythmic syndromes” beyond the arrhythmia definition: a comprehensive review. Int J Cardiovasc Imaging.

[30] Hannenhalli S, Putt ME, Gilmore JM, Wang J, Parmacek MS, Epstein JA (2006). Transcriptional genomics associates FOX transcription factors with human heart failure. Circulation.

[31] Barth AS, Kuner R, Buness A, Ruschhaupt M, Merk S, Zwermann L (2006). Identification of a common gene expression signature in dilated cardiomyopathy across independent microarray studies. J Am Coll Cardiol.

[32] Liu Y, Morley M, Brandimarto J, Hannenhalli S, Hu Y, Ashley EA (2015). RNA-Seq identifies novel myocardial gene expression signatures of heart failure. Genomics.

[33] Kanehisa M, Furumichi M, Sato Y, Kawashima M, Ishiguro-Watanabe M (2023). KEGG for taxonomy-based analysis of pathways and genomes. Nucleic Acids Res.

[34] Miličić D, Jakuš N, Fabijanović D (2018). Microcirculation and Heart failure. Curr Pharm Design.

[35] Dick SA, Epelman S (2016). Chronic heart failure and inflammation: what do we really know?. Circul Res.

[36] Nishida K, Otsu K (2017). Inflammation and metabolic cardiomyopathy. Cardiovascular Res.

[37] Dharmarajan K, Rich MW (2017). Epidemiology, pathophysiology, and prognosis of Heart failure in older adults. Heart Fail Clin.

[38] Yan C, Xu Z, Huang W (2021). Cellular Senescence affects Cardiac Regeneration and Repair in Ischemic Heart Disease. Aging and disease.

[39] Ghasempour G, Mohammadi A, Zamani-Garmsiri F, Soleimani AA, Najafi M (2022). Upregulation of TGF-β type II receptor in high glucose-induced vascular smooth muscle cells. Mol Biol Rep.

[40] Kazemi Fard T, Tavakoli S, Ahmadi R, Moradi N, Fadaei R, Mohammadi A (2020). Evaluation of IP10 and miRNA 296-a expression levels in Peripheral Blood mononuclear cell of coronary artery disease patients and controls. DNA Cell Biol.

[41] Mozaffarian D, Benjamin EJ, Go AS, Arnett DK, Blaha MJ, Cushman M (2016). Heart Disease and Stroke Statistics-2016 update: a Report from the American Heart Association. Circulation.

[42] Zhao X, Ren Y, Ren H, Wu Y, Liu X, Chen H (2021). The mechanism of myocardial fibrosis is ameliorated by myocardial infarction-associated transcript through the PI3K/Akt signaling pathway to relieve heart failure. J Int Med Res.

[43] Yan X, Cheng X, He X, Zheng W, Yuan X, Chen H (2018). HO-1 overexpressed mesenchymal stem cells ameliorate Sepsis-Associated Acute kidney Injury by activating JAK/stat3 pathway. Cell Mol Bioeng.

[44] Yuan FH, Chen YL, Zhao Y, Liu ZM, Nan CC, Zheng BL et al. microRNA-30a inhibits the liver cell proliferation and promotes cell apoptosis through the JAK/STAT signaling pathway by targeting SOCS-1 in rats with sepsis. J Cell Physiol. 2019;234(10):17839–53. 10.1002/jcp.28410 30972748

[45] Koitabashi N, Kass DA (2011). Reverse remodeling in heart failure–mechanisms and therapeutic opportunities. Nat reviews Cardiol.

[46] Rai A, Narisawa M, Li P, Piao L, Li Y, Yang G (2020). Adaptive immune disorders in hypertension and heart failure: focusing on T-cell subset activation and clinical implications. J Hypertens.

[47] Narikawa M, Umemura M, Tanaka R, Hikichi M, Nagasako A, Fujita T et al. Doxorubicin induces trans-differentiation and MMP1 expression in cardiac fibroblasts via cell death-independent pathways. PLoS One. 2019;14(9):e0221940. 10.1371/journal.pone.0221940 PMC6742217 31513610

[48] Mummidi S, Das NA, Carpenter AJ, Yoshida T, Yariswamy M, Mostany R et al. RECK suppresses interleukin-17/TRAF3IP2-mediated MMP-13 activation and human aortic smooth muscle cell migration and proliferation. J Cell Physiol. 2019;234(12):22242–59. 10.1002/jcp.28792 PMC7276214 31074012

[49] Renganathan V (2019). Overview of artificial neural network models in the biomedical domain. Bratisl Lek Listy.

[50] Yamashita R, Long J, Longacre T, Peng L, Berry G, Martin B (2021). Deep learning model for the prediction of microsatellite instability in colorectal cancer: a diagnostic study. Lancet Oncol.

[51] Li Y, Zhang Y, Zhang E, Chen Y, Wang Q, Liu K et al. Differential diagnosis of benign and malignant vertebral fracture on CT using deep learning. Eur Radiol. 2021;31(12):9612-9. 10.1007/s00330-021-08014-5 PMC8594282 33993335

[52] Elpeltagy M, Sallam H (2021). Automatic prediction of COVID- 19 from chest images using modified ResNet50. Multimedia tools and applications.

[53] Zhu X, Chen N, Liu L, Pu Q (2019). [An overview of the application of Artificial neural networks in Lung Cancer Research]. Zhongguo fei ai za zhi = Chinese. J lung cancer.

[54] Sandhu IK, Nair M, Shukla H, Sandhu SS (2015). Artificial neural network: as emerging Diagnostic Tool for breast Cancer. Int J Pharm Biol Sci.

[55] Schumann H, Holtz J, Zerkowski HR, Hatzfeld M (2000). Expression of secreted frizzled related proteins 3 and 4 in human ventricular myocardium correlates with apoptosis related gene expression. Cardiovascular Res.

[56] Charron S, Roubertie F, Benoist D, Dubes V, Gilbert SH, Constantin M (2015). Identification of region-specific myocardial gene expression patterns in a chronic swine model of repaired tetralogy of Fallot. PLoS ONE.

[57] Prohászka Z, Munthe-Fog L, Ueland T, Gombos T, Yndestad A, Förhécz Z (2013). Association of ficolin-3 with severity and outcome of chronic heart failure. PLoS ONE.

[58] Bai Z, Xu L, Dai Y, Yuan Q, Zhou Z (2021). ECM2 and GLT8D2 in human pulmonary artery hypertension: fruits from weighted gene co-expression network analysis. J Thorac disease.

[59] Cheng X, Liu Z, Liang W, Zhu Q, Wang C, Wang H (2023). ECM2, a prognostic biomarker for lower grade glioma, serves as a potential novel target for immunotherapy. Int J Biochem Cell Biol.

[60] Moestrup SK, Møller HJ (2004). CD163: a regulated hemoglobin scavenger receptor with a role in the anti-inflammatory response. Ann Med.

[61] Kowal K, Silver R, Sławińska E, Bielecki M, Chyczewski L, Kowal-Bielecka O (2011). CD163 and its role in inflammation. Folia Histochem Cytobiol.

[62] Kawamura K, Ishikawa K, Wada Y, Kimura S, Matsumoto H, Kohro T et al. Bilirubin from heme oxygenase-1 attenuates vascular endothelial activation and dysfunction. Arteriosclerosis, thrombosis, and vascular biology. 2005;25(1):155–60. 10.1161/01.ATV.0000148405.18071.6a 15499042

[63] Ptaszynska-Kopczynska K, Marcinkiewicz-Siemion M, Lisowska A, Waszkiewicz E, Witkowski M, Jasiewicz M (2016). Alterations of soluble TWEAK and CD163 concentrations in patients with chronic heart failure. Cytokine.

[64] Broch K, Ueland T, Yndestad A, Aukrust P, Gullestad L (2012). Heart failure biomarkers: focus on interleukin-1 receptor-like 1-based blood tests. Drugs of today (Barcelona Spain: 1998).

[65] Warbrick I, Rabkin SW (2019). Hypoxia-inducible factor 1-alpha (HIF-1α) as a factor mediating the relationship between obesity and heart failure with preserved ejection fraction. Obes reviews: official J Int Association Study Obes.

[66] Tomek J, Bub G. Hypertension-induced remodelling: on the interactions of cardiac risk factors. J Physiol. 2017;595(12):4027-36. 10.1113/JP273043 PMC5471416 28217927

[67] Cao M, Yuan W, Peng M, Mao Z, Zhao Q, Sun X et al. Role of CyPA in cardiac hypertrophy and remodeling. Biosci Rep. 2019;39(12). 10.1042/BSR20193190 PMC6928530 31825469

[68] Ludtmann MHR, Abramov AY (2018). Mitochondrial calcium imbalance in Parkinson’s disease. Neurosci Lett.

[69] Ahmed LA, Mohamed AF, Abd El-Haleim EA, El-Tanbouly DM (2021). Boosting akt pathway by Rupatadine modulates Th17/Tregs balance for attenuation of Isoproterenol-Induced Heart failure in rats. Front Pharmacol.

[70] Soleimani AA, Ghasmpour G, Mohammadi A, Gholizadeh M, Abkenar BR, Najafi M (2022). Focal adhesion kinase-related pathways may be suppressed by metformin in vascular smooth muscle cells in high glucose conditions. Endocrinol diabetes metabolism.

[71] Wallner FK, Hultqvist Hopkins M, Lindvall T, Olofsson P, Tilevik A (2017). Cytokine correlation analysis based on drug perturbation. Cytokine.

[72] Patente TA, Pinho MP, Oliveira AA, Evangelista GCM, Bergami-Santos PC, Barbuto JAM (2018). Human dendritic cells: their heterogeneity and clinical application potential in Cancer Immunotherapy. Front Immunol.

[73] Kologrivova I, Shtatolkina M, Suslova T, Ryabov V (2021). Cells of the Immune System in Cardiac Remodeling: main players in resolution of inflammation and repair after myocardial infarction. Front Immunol.

[74] Kumrić M, Kurir TT, Borovac JA, Božić J. The Role of Natural Killer (NK) Cells in Acute Coronary Syndrome: A Comprehensive Review. Biomolecules. 2020;10(11):1514. 10.3390/biom10111514 PMC7694449 33167533

[75] Wolf D, Gerhardt T, Winkels H, Michel NA, Pramod AB, Ghosheh Y (2020). Pathogenic autoimmunity in atherosclerosis evolves from initially protective apolipoprotein B(100)-Reactive CD4(+) T-Regulatory cells. Circulation.

